# Semi-quantitative assessment of paraspinal muscle fat infiltration in single-level C5–6 cervical spondylosis using gray-level thresholding on conventional MRI: associations with disc degeneration and posture-related segmental angular change

**DOI:** 10.3389/fmed.2026.1889821

**Published:** 2026-08-26

**Authors:** Yifei Li, Liang Zhao, Wei Zhang, Xiubo Ge, Haitao Lu, Haoran Shi, Haiyang Yu

**Affiliations:** 1 Department of Orthopedics, Fuyang People’s Hospital Affiliated to Anhui Medical University (Fuyang People’s Hospital), Fuyang, Anhui, China; 2 Clinical Research Center for Spinal Deformity of Anhui Province, Fuyang, Anhui, China

**Keywords:** cervical spondylosis, cross-sectional area, fat infiltration, paraspinal muscles, Pfirrmann grade, posture-related angular change, semi-quantitative MRI

## Abstract

**Background:**

Cervical spondylosis at C5–6 is common, but conventional imaging may not fully capture paraspinal muscle composition or posture-related segmental angular behavior. This study assessed layer-specific paraspinal muscle fat infiltration (FI) and cross-sectional area (CSA) on conventional MRI and examined their associations with C5–6 disc degeneration and posture-related angular change (PAC).

**Methods:**

In this retrospective cross-sectional study, 94 patients with single-level C5–6 cervical spondylosis who underwent cervical MRI and standing lateral radiography between January 2023 and December 2025 were included. At the C5–6 disc level, bilateral splenius capitis (SPL), semispinalis capitis (SCap), and combined multifidus plus semispinalis cervicis (MF + SCer) were manually segmented. FI was calculated as a semi-quantitative area percentage using a fixed 8-bit gray-level threshold (79–255), and CSA was measured on calibrated axial T2-weighted images. PAC was defined as the absolute difference between standing radiographic and supine MRI local C5–6 Cobb angles; patients were classified as low-PAC/relatively stiff (|PAC| ≤ 5°) or high-PAC/relatively mobile (|PAC| > 5°).

**Results:**

FI in all three muscle groups increased with higher Pfirrmann grades, whereas CSA did not differ significantly across grades. Pfirrmann grade was independently associated with FI in the SPL, SCap, and MF + SCer after adjustment for age and sex. Compared with the high-PAC group, the low-PAC group showed higher FI in the SPL, SCap, and MF + SCer, and these differences remained significant after adjustment for age, sex, BMI, and Pfirrmann grade. CSA did not differ significantly between PAC phenotypes.

**Conclusions:**

In patients with single-level C5–6 cervical spondylosis, paraspinal muscle alteration was characterized primarily by increased FI, whereas CSA reduction was not evident. Compared with CSA, semi-quantitative FI measured on conventional MRI may be more sensitive for detecting observable changes in muscle composition. Higher FI in the superficial and intermediate posterior extensor muscles was associated with a low-PAC/relatively stiff phenotype. Combining PAC with paraspinal muscle FI may provide supplementary imaging information on segmental angular differences between weight-bearing and non-weight-bearing positions; however, the clinical relevance of this combined assessment requires prospective validation using quantitative fat imaging and clinical or functional outcomes.

## 1 Introduction

Cervical spondylosis is a common degenerative spinal disorder characterized by cervical disc degeneration and secondary changes in adjacent joint structures. These changes may involve surrounding tissues and lead to a range of clinical symptoms (1–4). In current practice, imaging assessment mainly relies on radiography, CT, and MRI to identify structural changes, such as disc herniation, osteophyte formation, spinal canal or foraminal narrowing, and neural compression. Sagittal alignment parameters are also used to evaluate overall and segmental cervical alignment. However, patients with similar degrees of structural degeneration may still differ in segmental stability, postural control, and functional limitation. This suggests that static structural imaging alone may not fully reflect the functional status of the affected segment (4, 5).

The paraspinal muscles play an important role in maintaining dynamic balance and postural stability of the cervical spine (6–9). Anatomically, they can be broadly divided into superficial and intermediate muscles involved in movement and deeper muscles involved in stabilization. In recent years, cross-sectional area (CSA) measured on standardized axial MRI and semi-quantitative fat infiltration (FI), calculated as the percentage area of high-signal fat-related pixels using threshold-based segmentation, have increasingly been used to assess paraspinal muscle degeneration and related functional impairment (6, 10–17). The study by Ornowski et al. (11) provides methodological support for estimating paraspinal muscle fat infiltration using thresholding on conventional MRI. Because that study did not specifically focus on the C5–6 cervical level, we used it as a methodological reference and improved reproducibility through standardized region-of-interest definition and reliability testing. In clinical imaging, the local Cobb angle may differ between supine MRI, which is acquired without weight bearing, and standing lateral radiography, which is acquired under weight-bearing conditions. In this study, this difference was defined as posture-related angular change (PAC). PAC describes the angular difference of the affected segment across imaging positions and loading conditions, but it should not be regarded as a substitute for dynamic flexion-extension radiography in assessing true segmental mobility (18–23). Previous studies have often assessed the posterior cervical muscles as a single muscle group. Less attention has been paid to layer-specific degeneration patterns in muscles such as the splenius capitis (SPL), semispinalis capitis (SCap), and multifidus plus semispinalis cervicis (MF + SCer) in the setting of single-level disease. Studies combining paraspinal muscle imaging parameters with PAC phenotypes also remain limited.

Based on this rationale, the present study enrolled patients with single-level C5–6 cervical spondylosis. Standardized axial MRI was used to measure the CSA of the SPL, SCap, and MF + SCer, and gray-level thresholding was applied to calculate semi-quantitative FI as an area percentage. Disc degeneration was evaluated using the Pfirrmann grading system. Although this system was originally developed for grading lumbar disc degeneration (24), it was applied in this study to describe morphological degeneration at the C5–6 cervical level, with support from previous studies on cervical disc degeneration (25–28). To complement static structural assessment from the perspective of posture-related angular change, we used the absolute difference in the C5–6 local Cobb angle between standing lateral radiography and supine MRI as an operational measure. A prespecified threshold of 5° was used to classify patients into a low-PAC/relatively stiff phenotype and a high-PAC/relatively mobile phenotype (22, 29). By jointly analyzing layer-specific paraspinal muscle imaging parameters, disc degeneration, and PAC phenotype, this study aimed to identify muscle degeneration features with potential imaging relevance in single-level C5–6 cervical spondylosis and to provide additional information for refined imaging assessment.

## 2 Materials and methods

### 2.1 Study population

This single-center retrospective cross-sectional study was approved by the Ethics Committee of our institution (approval Medical Ethics Review ([2024]) No. 206) and was conducted in accordance with the Declaration of Helsinki. Because the study was based on existing clinical imaging and medical records and involved no additional intervention in patient care, the requirement for informed consent was waived by the Ethics Committee. Patients with single-level C5–6 cervical spondylosis who were treated at our institution between January 2023 and December 2025 and underwent baseline cervical MRI and standing lateral cervical radiography were retrospectively screened. A total of 94 patients were finally included. The inclusion criteria were as follows: (1) complete baseline standardized cervical radiographic and MRI data, with imaging evidence of nerve root or spinal cord compression mainly caused by soft compression at the C5–6 level, such as disc herniation; (2) typical clinical symptoms and signs of cervical radiculopathy or myelopathy consistent with C5–6 segmental pathology; and (3) no physical therapy, acupuncture, local injection, or other treatments that could affect paraspinal muscle morphology or tone within 4 weeks before imaging. The exclusion criteria were as follows: (1) congenital spinal dysplasia or severe spinal deformity, such as scoliosis or kyphosis; (2) specific cervical infection, ossification of the posterior longitudinal ligament, intraspinal tumor, cervical trauma, or metabolic bone disease; (3) autoimmune diseases affecting systemic joints or cervical curvature, such as ankylosing spondylitis or rheumatoid arthritis; (4) previous cervical surgery or revision surgery; (5) severe dysfunction of major organs, including the heart, liver, or kidneys, resulting in inability to complete the examination, or incomplete clinical data; and (6) severe MRI artifacts that prevented clear identification and delineation of paraspinal muscle boundaries.

### 2.2 Data acquisition and measurement methods

#### 2.2.1 Cervical MRI protocol

MRI was performed using a 3.0-T MRI scanner (Ingenia, Philips Healthcare). Patients were positioned supine and head first, with routine head support for stabilization. Images were acquired using a combined head and neck coil. The MRI protocol included mid-sagittal T1-weighted imaging, sagittal T2-weighted imaging, and axial T2-weighted imaging. Axial T2-weighted images used for paraspinal muscle fat infiltration (FI) and cross-sectional area (CSA) analysis were acquired using a two-dimensional fast spin-echo/turbo spin-echo sequence without fat suppression. The main imaging parameters were as follows: repetition time, 3,000–4,500 ms; echo time, 90–120 ms; echo train length/TSE factor, 15–25; slice thickness, 3.0–4.0 mm; interslice gap, 0.3–0.5 mm; field of view, 160 mm × 160 mm to 180 mm × 180 mm; acquisition matrix, 256 × 256 to 320 × 256; reconstruction matrix, 512 × 512; number of excitations, 1–2; and acquisition time, approximately 2.0–3.5 min. All C5–6 axial T2-weighted images used for paraspinal muscle analysis were exported from the PACS as 8-bit PNG images using uniform window width, window level, and display settings, with scale information retained. After import into ImageJ/Fiji, image scale calibration was performed according to the PACS scale.

#### 2.2.2 Standing lateral cervical radiography protocol

Standardized standing lateral cervical radiographs were obtained using the digital radiography system at our institution (Siemens, Germany). During image acquisition, patients stood naturally with both arms relaxed at their sides. The auriculo-nasal line was kept parallel to the floor to maintain a neutral head and neck position. The central x-ray beam was aligned with the C4 vertebral body. The imaging field extended superiorly to the external occipital protuberance and inferiorly to include the C7/T1 interspace.

#### 2.2.3 Assessment of cervical disc degeneration

Supine mid-sagittal T2-weighted MR images were reviewed in the PACS. The degree of C5–6 disc degeneration was morphologically graded using the Pfirrmann grading system (24–28). Although the Pfirrmann grading system was originally developed for lumbar disc degeneration, it was used in this study as an ordinal reference commonly adopted in cervical degeneration research, rather than as the sole gold standard for cervical disc degeneration. Because Pfirrmann grade I is usually observed in younger individuals without obvious degeneration, the patients included in this study were mainly distributed across grades II–V. The grading was independently performed by three physicians with experience in spinal imaging assessment. They were blinded to the muscle measurements and PAC grouping. Any disagreement was resolved by discussion until consensus was reached.

#### 2.2.4 Measurement of cervical paraspinal muscle CSA and FI and reliability assessment

The central axial MRI slice at the C5–6 disc level was selected as the measurement plane. All images were calibrated using the scale information retained in the PACS, and cross-sectional area (CSA) was expressed in mm^2^. Three trained observers, blinded to clinical data, Pfirrmann grade, and PAC phenotype, independently outlined the regions of interest (ROIs) for the bilateral splenius capitis (SPL), semispinalis capitis (SCap), and combined multifidus plus semispinalis cervicis (MF + SCer) muscles according to standardized anatomical boundaries. The SPL was traced along its lateral fascial border. The MF + SCer was defined as the deepest muscle group adjacent to both sides of the spinous process and lamina. The SCap was defined as the muscle layer located between the SPL and MF + SCer. Its lateral boundary was the lateral fascial line of the posterior muscle group, its medial boundary was the posterior projection of the lateral edge of the lamina, its superficial boundary was the visible fascial or fat plane separating it from the SPL, and its deep boundary was the interface with the MF + SCer. When local boundaries were unclear, the lateral fascial line, the projected edge of the lamina, and the relative anatomical position of the SCap to the SPL and MF + SCer were used to ensure consistent delineation (6, 14, 15). Left and right measurements were averaged for each muscle before statistical analysis.

**Figure 1:**
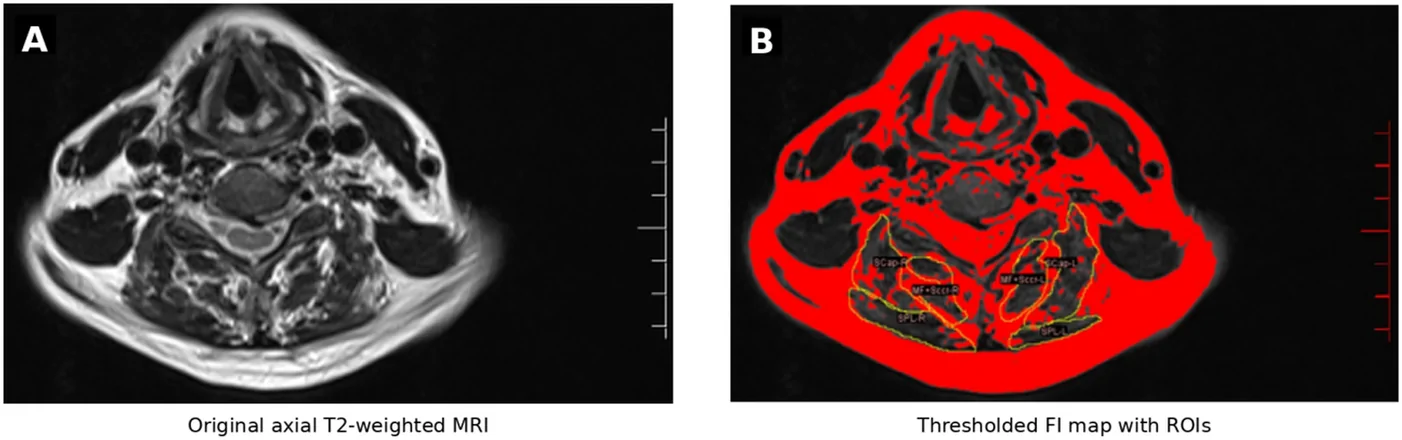
ROI delineation and threshold-based fat infiltration (FI) measurement workflow at C5–6. **(A)** Original axial T2-weighted MRI at the central C5–6 disc level. **(B)** Thresholded FI map after applying the fixed 8-bit gray-level threshold of 79–255 within the manually delineated bilateral ROIs for the splenius capitis (SPL), semispinalis capitis (SCap), and combined multifidus plus semispinalis cervicis (MF + SCer). FI (%) was calculated as threshold-positive high-signal fat-related pixel area divided by total ROI area and multiplied by 100%. This method yields a semi-quantitative area percentage, not a quantitative fat fraction.

After ROI delineation, semi-quantitative fat infiltration (FI) analysis was performed using ImageJ/Fiji. Because gray-level values on conventional T2-weighted images can be affected by imaging parameters, coil configuration, field inhomogeneity, window settings, and image export procedures, FI in this study was defined as the percentage area of high-signal fat-related pixels obtained through a standardized image-processing workflow. All exported images were 8-bit grayscale images with gray-level values ranging from 0 to 255. Based on the gray-level distribution of representative ROIs for subcutaneous fat, muscle tissue, and intermuscular fat in the preliminary analysis, together with threshold visualization in ImageJ/Fiji, the fixed gray-level threshold for the 8-bit images was set at 79–255. This threshold was used only for semi-quantitative segmentation of high-signal fat-related pixels under the standardized image export workflow of this study, and should not be interpreted as a quantitative fat fraction. The same threshold was applied to all cases, all muscle groups, and all observers. High-signal pixels within this threshold range were defined as fat-related pixels. FI (%) was calculated as follows: area of high-signal fat-related pixels within the ROI/total ROI area × 100%. Therefore, FI in this study represents a semi-quantitative area percentage derived from gray-level thresholding on conventional T2-weighted images, rather than a proton density fat fraction measured using Dixon or IDEAL-IQ sequences. The segmentation results were checked against the original images by the observers, and high-signal areas outside the ROI, vessels, cortical bone margins, and obvious artifacts were excluded. All measurements were performed by three independent observers. One observer repeated the measurements after 1 week. Interobserver and intraobserver reliability were assessed using the intraclass correlation coefficient (ICC), with an ICC greater than 0.75 considered to indicate good reproducibility (30, 31). A representative workflow of ROI delineation and fixed-threshold FI segmentation is shown in Figure 1.

#### 2.2.5 Assessment of posture-related segmental angular change and PAC phenotype classification

**Figure 2:**
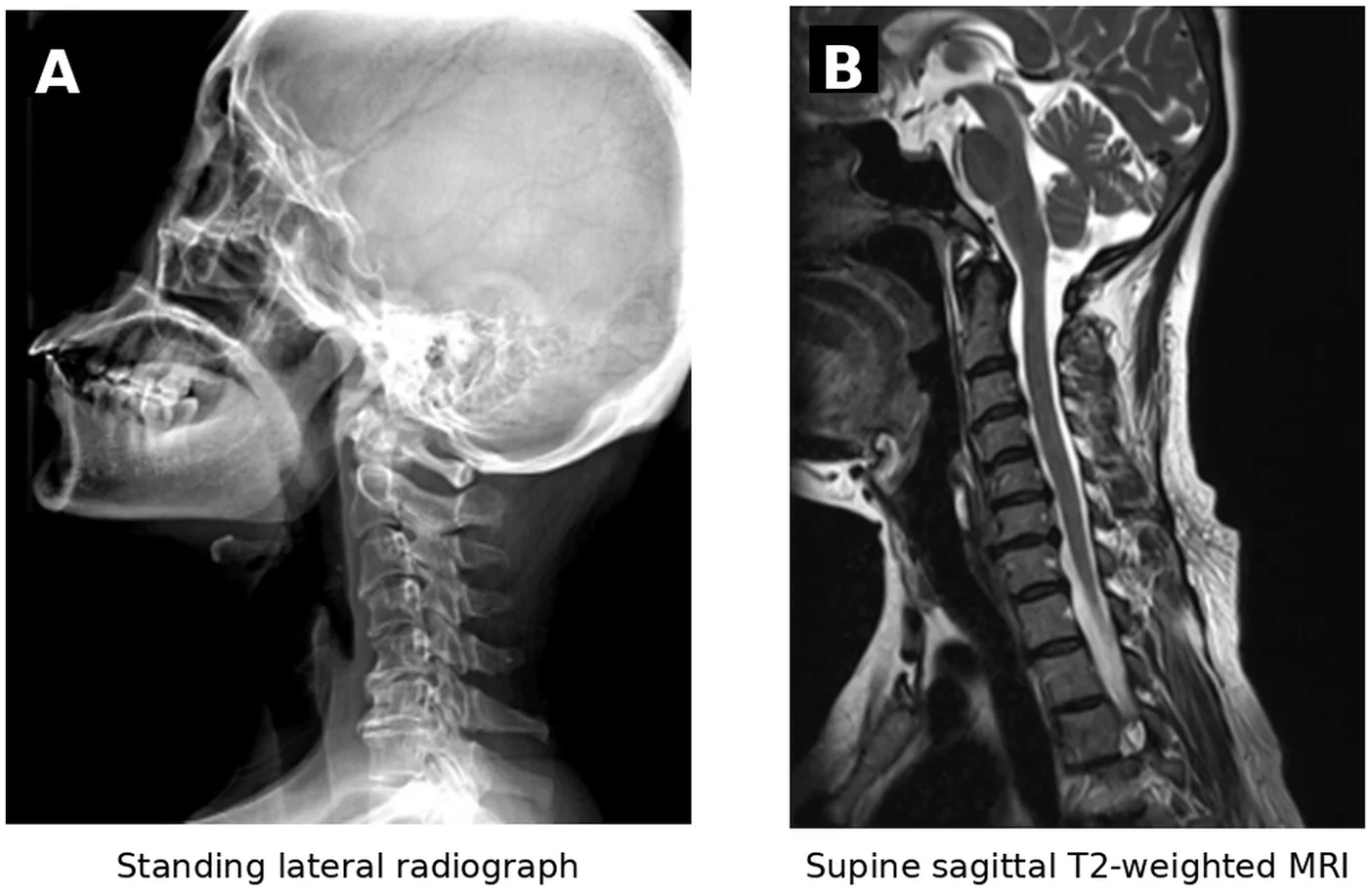
Representative assessment of posture-related angular change (PAC) at C5–6. A: Standing lateral cervical radiograph. B: Supine mid-sagittal T2-weighted MRI. The local C5–6 Cobb angle was measured on each image using tangent lines to the inferior endplates of C5 and C6. Absolute PAC was defined as |standing radiographic Cobb angle—supine MRI Cobb angle|. Patients with |PAC| ≤ 5° were classified as low-PAC/relatively stiff, whereas those with |PAC| > 5° were classified as high-PAC/relatively mobile. PAC is an operational posture-related angular measure and is not equivalent to dynamic flexion-extension mobility.

The local C5–6 Cobb angle was measured on supine mid-sagittal T2-weighted cervical MRI and standing lateral cervical radiographs. The angle was defined as the angle between the tangent lines of the inferior endplates of the C5 and C6 vertebral bodies. The absolute value of the difference in local Cobb angle between the two positions was defined as absolute posture-related angular change: absolute PAC = |standing radiographic Cobb angle − supine MRI Cobb angle| (18–23). PAC reflects the local angular difference at C5–6 between different imaging positions and loading conditions. It should not be considered equivalent to true segmental mobility measured on dynamic flexion-extension radiographs. In this study, PAC was used as an operational measure of posture-related segmental angular change. Considering measurement error and clinical practicality, a prespecified threshold of 5° was used for classification (22, 29). Patients with |PAC| ≤ 5° were assigned to the low-PAC/relatively stiff phenotype group, whereas those with |PAC| > 5° were assigned to the high-PAC/relatively mobile phenotype group. All angular measurements were independently performed by three senior spine surgeons. One observer repeated the measurements after 1 week. The ICC was used to assess the reliability of local Cobb angle measurements on standing radiographs and supine MRI, and the Kappa coefficient was used to evaluate agreement in PAC phenotype classification (32–34). A representative example of PAC assessment using standing radiography and supine MRI is shown in Figure 2.

### 2.3 Statistical analysis

Statistical analyses were performed using SPSS version 25.0. Continuous variables were tested for normality and homogeneity of variance before group comparisons. Categorical variables were presented as counts and compared using the chi-square test or Fisher's exact test, as appropriate. Continuous variables with a normal or approximately normal distribution were presented as mean ± standard deviation. Comparisons between two groups were performed using the independent-samples t test, and comparisons among multiple groups were performed using one-way analysis of variance. When the assumption of equal variances was not met, Welch's correction was applied, followed by Games–Howell *post hoc* tests. Non-normally distributed continuous variables were presented as median with interquartile range. The Mann–Whitney *U* test was used for two-group comparisons, and the Kruskal–Wallis test was used for comparisons among more than two groups. Pfirrmann grade was treated as an ordinal variable. In addition to group comparisons, Spearman correlation analysis was used to evaluate the ordinal trends between increasing Pfirrmann grade and FI or CSA. Because the sample size for Pfirrmann grade *V* was small (*n* = 6), *post hoc* comparisons and stratified analyses involving grade *V* were interpreted as exploratory findings. SPL FI was prespecified as the primary muscle-related outcome, whereas SCap FI, MF + SCer FI, and CSA-related analyses were considered secondary or exploratory outcomes. Given that multiple muscle groups and multiple comparisons were analyzed, exploratory findings were interpreted cautiously, with attention to the direction and magnitude of effects where applicable. Exploratory findings were not used to infer causality. Reliability of continuous measurements was assessed using the ICC, and agreement in PAC phenotype classification was assessed using the Kappa coefficient (30–34). Multiple linear regression was used to identify factors independently associated with cervical paraspinal muscle FI and CSA. Sex, age, and Pfirrmann grade were included as covariates in the primary regression models for paraspinal muscle FI and CSA. Because absolute CSA is body-size dependent and BMI is an indirect anthropometric surrogate that does not distinguish lean mass from adiposity, BMI was not included in the primary CSA models. Therefore, CSA-related findings were interpreted cautiously with respect to potential body-size effects. In an exploratory sensitivity analysis, the association between PAC phenotype and FI was further evaluated using separate linear regression models for SPL FI, SCap FI, and MF + SCer FI, with the high-PAC/relatively mobile group as the reference category and age, sex, BMI, and Pfirrmann grade included as covariates. *P* < 0.05 was considered statistically significant.

## 3 Results

### 3.1 Patient characteristics and measurement reliability

A total of 94 patients with single-level C5–6 cervical spondylosis were included. According to Pfirrmann grade, 20 patients were classified as grade II, 41 as grade III, 27 as grade IV, and 6 as grade V (Table 1). Based on PAC phenotype, 49 patients were assigned to the low-PAC/relatively stiff group and 45 to the high-PAC/relatively mobile group (Table 2). Sex, age, and body mass index did not differ significantly among Pfirrmann grade groups (*P* = 0.077, 0.151, and 0.877, respectively; Table 1). These variables also showed no significant differences between the two PAC phenotype groups (*P* = 0.298, 0.811, and 0.683, respectively; Table 2).

**Table 1:** Comparison of baseline characteristics Among patients with different Pfirrmann grades.

Variable	Grade II (*n* = 20)	Grade III (*n* = 41)	Grade IV (*n* = 27)	Grade *V* (*n* = 6)	*P*
Male/Female	14/6	21/20	9/18	2/4	0.077
Age, years	54.650 ± 4.534	57.366 ± 10.319	58.519 ± 6.818	63.167 ± 10.048	0.151
BMI, kg/m^2^	25.040 ± 2.052	24.785 ± 2.389	25.167 ± 2.429	25.400 ± 1.682	0.877

Data are presented as *n* or mean ± standard deviation. BMI, body mass index.

**Table 2:** Comparison of baseline characteristics between patients with different PAC phenotypes.

Variable	Low-PAC/Relatively Stiff Group (*n* = 49)	High-PAC/Relatively Mobile Group (*n* = 45)	*P*
Male/Female	27/22	19/26	0.298
Age, years	57.000 (53.000, 62.000)	57.000 (53.000, 62.000)	0.811
BMI, kg/m^2^	24.896 ± 2.243	25.089 ± 2.322	0.683
Pfirrmann grade (II/III/IV/V)	8/20/16/5	12/21/11/1	0.235

Data are presented as *n*, median (interquartile range), or mean ± standard deviation, as appropriate. BMI, body mass index; PAC, posture-related angular change. Categorical variables with small expected cell counts were compared using Fisher's exact test.

Measurements of FI and CSA for the SPL, SCap, and MF + SCer at the C5–6 level showed good reliability, with interobserver ICCs ranging from 0.833 to 0.880 and intraobserver ICCs ranging from 0.811 to 0.877 (Table 3). The reliability of local C5–6 Cobb angle measurements was also good on both standing radiographs and supine MRI, with interobserver ICCs of 0.873 and 0.871, respectively, and intraobserver ICCs of 0.972 and 0.940, respectively (Table 3). After phenotype classification using the 5° threshold for |PAC|, overall agreement among the three observers was excellent (Fleiss’ *κ* = 0.943, 95% CI: 0.885–0.986; Table 3).

**Table 3:** Reliability analysis of imaging measurements.

Parameter	Intraobserver ICC/*κ*	95%CI	Interobserver ICC/*κ*	95%CI
SPL FI (%)	0.828	0.731–0.885	0.843	0.784–0.882
SPL CSA (mm^2^)	0.815	0.746–0.868	0.834	0.775–0.877
SCap FI(%)	0.877	0.809–0.920	0.865	0.801–0.904
SCap CSA(mm^2^)	0.811	0.720–0.869	0.841	0.783–0.877
MF + SCer FI(%)	0.825	0.760–0.873	0.833	0.782–0.872
MF + SCer CSA(mm^2^)	0.831	0.758–0.877	0.880	0.833–0.909
C5–6 Cobb angle on standing radiographs	0.972	0.964–0.977	0.873	0.836–0.899
C5–6 Cobb angle on supine MRI	0.940	0.907–0.960	0.871	0.832–0.899
Agreement in PAC phenotype classification	0.893*	0.790–0.978	0.943†	0.885–0.986

ICC, intraclass correlation coefficient; CI, confidence interval; FI, fat infiltration; CSA, cross-sectional area; SPL, splenius capitis; SCap, semispinalis capitis; MF + SCer, multifidus plus semispinalis cervicis; PAC, posture-related angular change.

* Indicates Cohen's *κ* for repeated measurements by Observer 1.

† Indicates overall Fleiss’ *κ* among the three observers.

### 3.2 Comparison of paraspinal muscle FI and CSA across Pfirrmann grades

**Figure 3:**
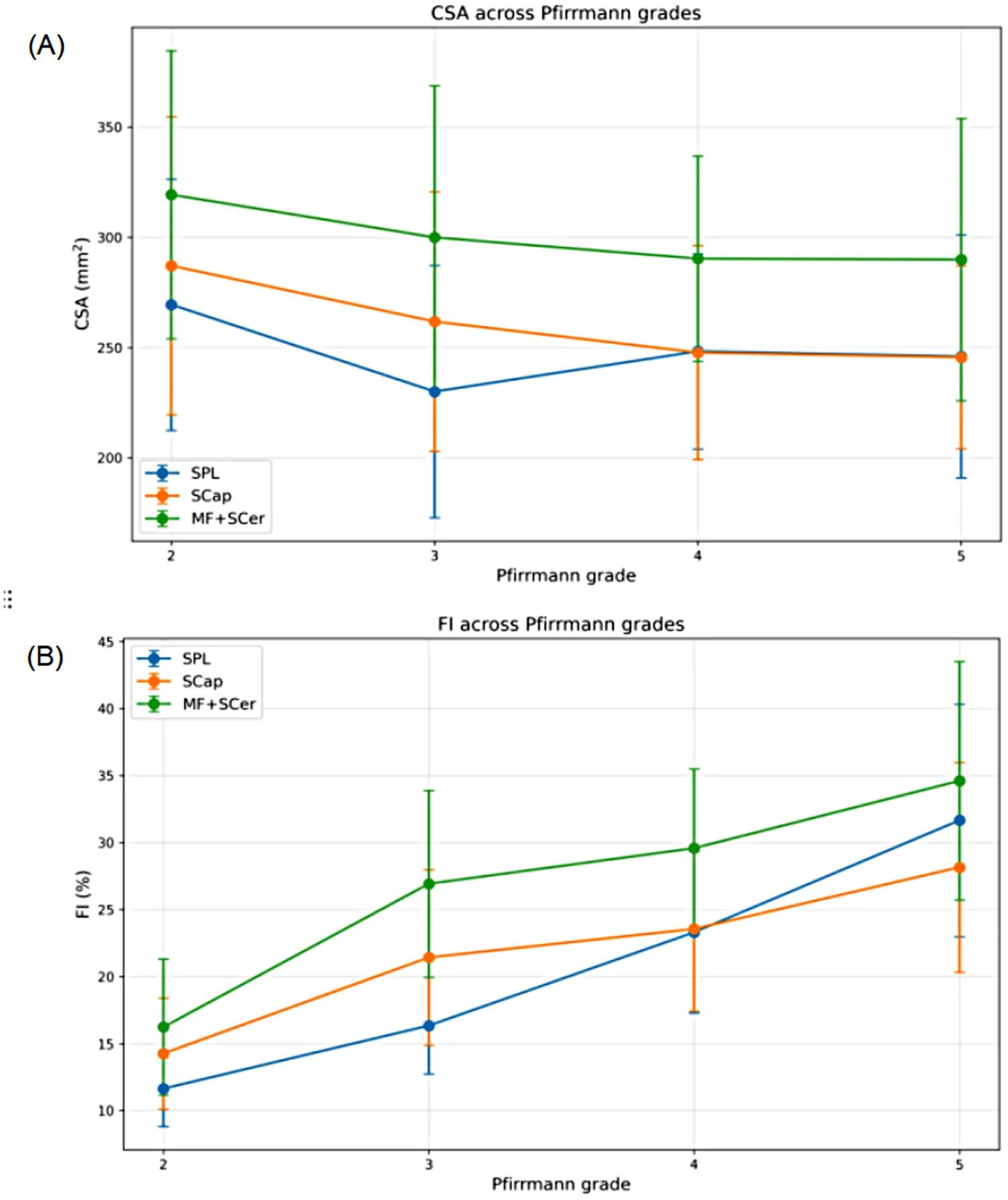
Changes in C5–6 paraspinal muscle cross-sectional area and fat infiltration across Pfirrmann grades. **(A)** Cross-sectional area (CSA) of the C5–6 paraspinal muscles across Pfirrmann grades. **(B)** Fat infiltration (FI) of the C5–6 paraspinal muscles increased with higher Pfirrmann grades. Error bars indicate ± standard deviation.

FI in the SPL, SCap, and MF + SCer increased progressively with higher Pfirrmann grades, whereas CSA did not differ significantly among the grade groups for any muscle group. Specifically, SPL FI increased from 11.635% ± 2.836% in grade II to 31.655% ± 8.672% in grade V (*F* = 32.934, *P* < 0.001; Table 4 and Figure 3B). SCap FI increased from 14.257% ± 4.145% to 28.153% ± 7.835% (*F* = 12.609, *P* < 0.001; Table 4 and Figure 3B), and MF + SCer FI increased from 16.230% ± 5.083% to 34.603% ± 8.897% (*F* = 21.952, *P* < 0.001; Table 4 and Figure 3B). In contrast, CSA of the SPL, SCap, and MF + SCer showed no significant differences across Pfirrmann grades (*P* = 0.066, 0.119, and 0.438, respectively; Table 4 and Figure 3A).

**Table 4:** Comparison of C5–6 paraspinal muscle FI and CSA across Pfirrmann grades.

Parameter	Grade II	Grade III	Grade IV	Grade *V*	*F*	*P*
SPL FI (%)	11.635 ± 2.836	16.339 ± 3.597	23.300 ± 6.026	31.655 ± 8.672	32.934	<0.001
SCap FI (%)	14.257 ± 4.145	21.424 ± 6.551	23.553 ± 6.137	28.153 ± 7.835	12.609	<0.001
MF + SCer FI (%)	16.230 ± 5.083	26.924 ± 6.971	29.578 ± 5.897	34.603 ± 8.897	21.952	<0.001
SPL CSA (mm^2^)	269.481 ± 56.983	230.066 ± 57.222	248.321 ± 44.306	246.008 ± 55.043	2.483	0.066
SCap CSA (mm^2^)	287.098 ± 67.618	261.812 ± 58.873	247.727 ± 48.535	245.610 ± 41.474	2.002	0.119
MF + SCer CSA (mm^2^)	319.332 ± 65.343	299.956 ± 68.786	290.330 ± 46.592	289.897 ± 63.936	0.913	0.438

FI, fat infiltration; CSA, cross-sectional area; SPL, splenius capitis; SCap, semispinalis capitis; MF + SCer, multifidus plus semispinalis cervicis. Data are presented as mean ± standard deviation.

### 3.3 *Post hoc* multiple comparisons

Post hoc analysis showed significant differences in SPL FI between grade II and grades III, IV, and V, as well as between grade III and grades IV and V (all *P* < 0.05; Table 5). No significant difference was observed between grades IV and V (*P* = 0.212; Table 5). For SCap FI and MF + SCer FI, significant differences were found only between grade II and grades III, IV, and V (all *P* < 0.05; Table 5). The remaining pairwise comparisons were not statistically significant (all *P* > 0.05; Table 5).

**Table 5:** Pairwise comparisons of FI Among Pfirrmann grades using the games–howell test.

Parameter	Grade (I)	Grade (J)	Mean difference (I−J)	*P*
SPL FI	II	III	−4.704*	<0.001
SPL FI	II	IV	−11.665*	<0.001
SPL FI	II	V	−20.020*	0.008
SPL FI	III	IV	−6.962*	<0.001
SPL FI	III	V	−15.316*	0.026
SPL FI	IV	V	−8.355	0.212
SCap FI	II	III	−7.167*	<0.001
SCap FI	II	IV	−9.295*	<0.001
SCap FI	II	V	−13.896*	0.024
SCap FI	III	IV	−2.129	0.528
SCap FI	III	V	−6.729	0.283
SCap FI	IV	V	−4.600	0.567
MF + SCer FI	II	III	−10.693*	<0.001
MF + SCer FI	II	IV	−13.347*	<0.001
MF + SCer FI	II	V	−18.373*	0.011
MF + SCer FI	III	IV	−2.654	0.339
MF + SCer FI	III	V	−7.679	0.278
MF + SCer FI	IV	V	−5.026	0.584

FI, fat infiltration; SPL, splenius capitis; SCap, semispinalis capitis; MF + SCer, multifidus plus semispinalis cervicis.

* Indicates a statistically significant pairwise difference.

### 3.4 Correlation and multiple linear regression analyses

**Figure 4:**
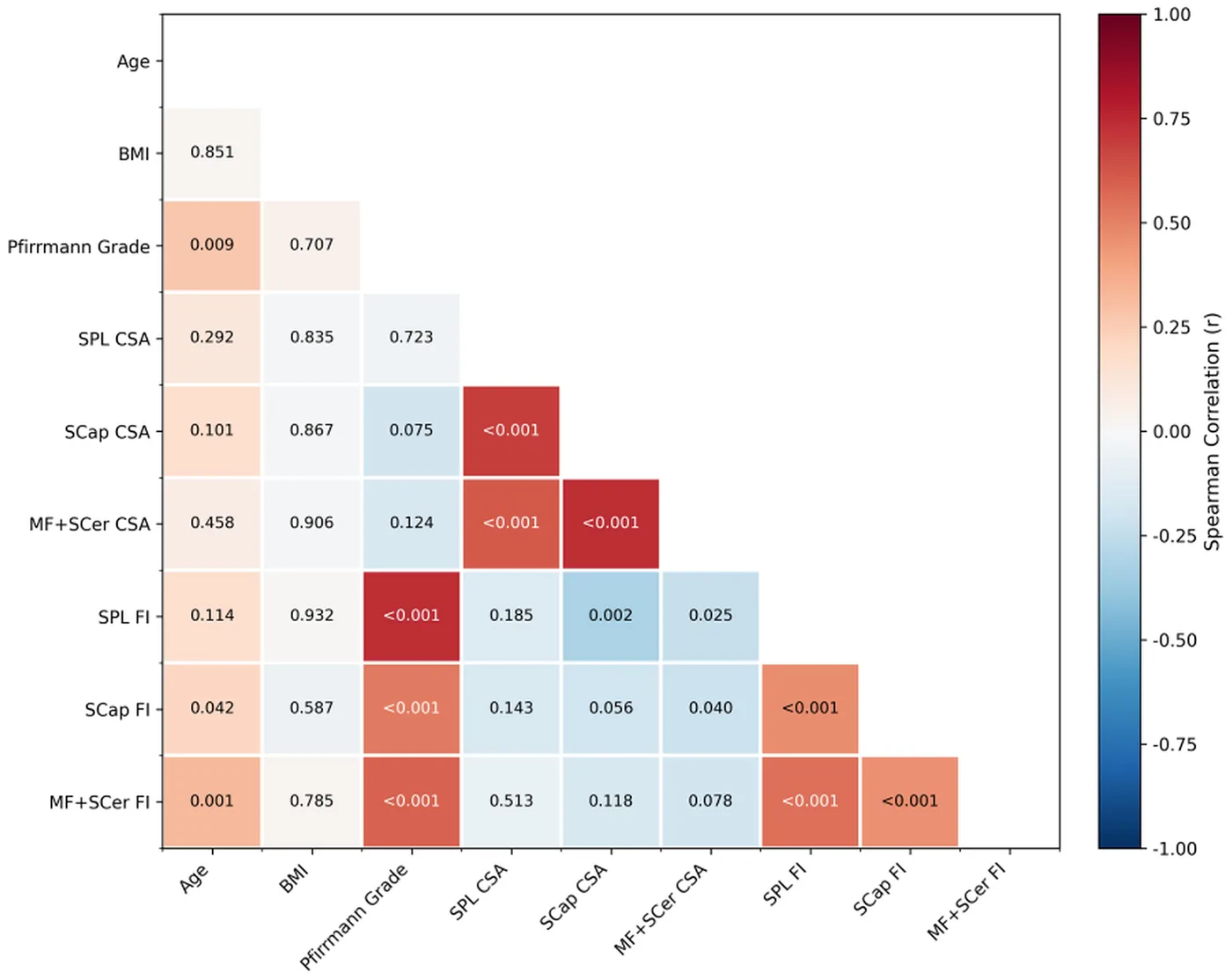
Spearman correlation heatmap of the study variables. Cell colors indicate Spearman correlation coefficients, and the values shown in the cells indicate *P* values.

Spearman correlation analysis showed that Pfirrmann grade was significantly and positively correlated with FI in the SPL, SCap, and MF + SCer. The strongest correlation was observed for SPL FI (*r* = 0.739, *P* < 0.001; Figure 4), while MF + SCer FI and SCap FI showed moderate correlations with Pfirrmann grade (*r* = 0.587 and 0.519, respectively; both *P* < 0.001; Figure 4). In contrast, CSA of the three muscle groups was not significantly correlated with Pfirrmann grade (*P* = 0.723, 0.075, and 0.124, respectively; Figure 4).

**Figure 5:**
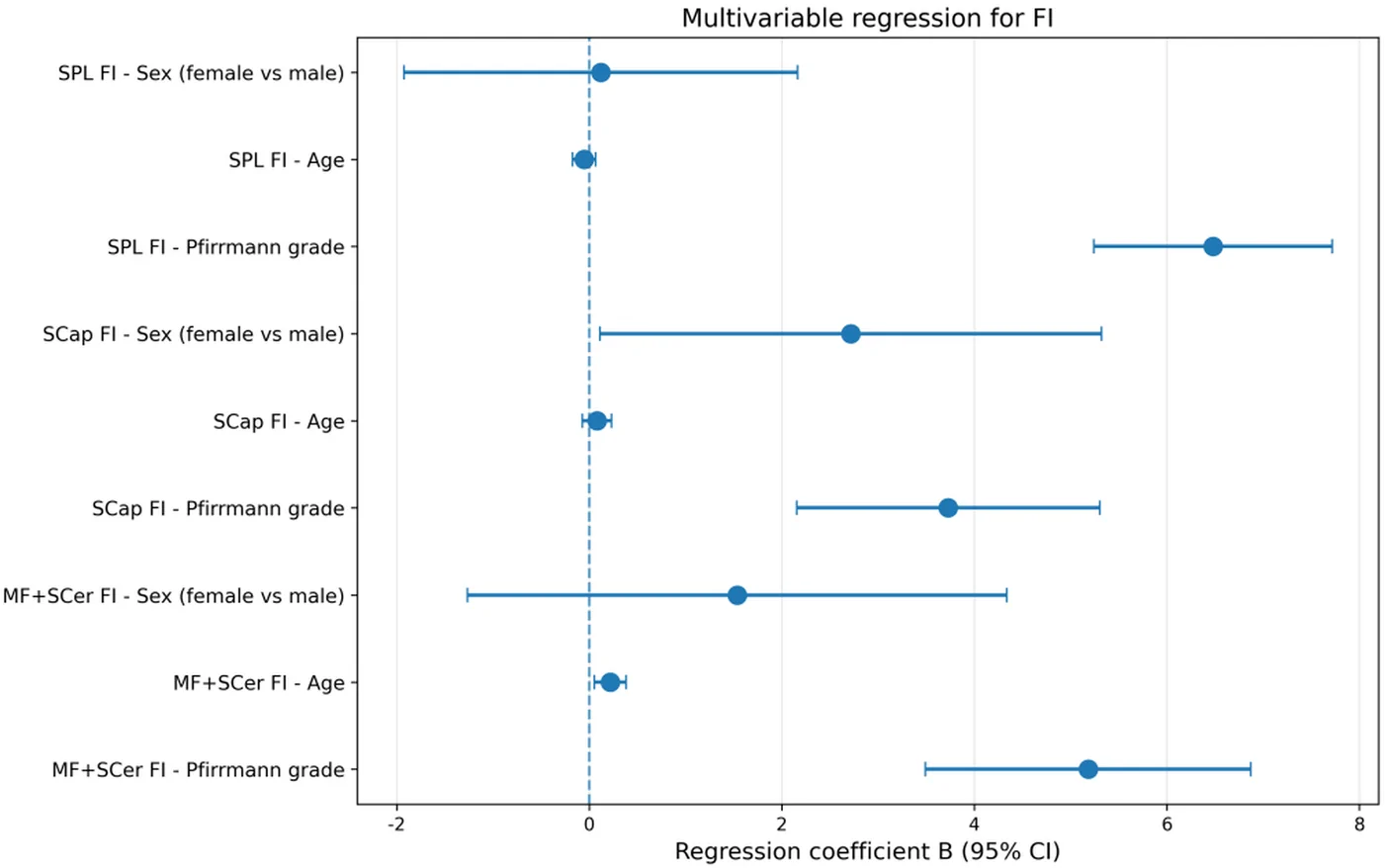
Forest plot of the multiple linear regression results for paraspinal muscle FI. Dots represent the regression coefficient **(B)**, and horizontal lines represent the 95% confidence interval (CI).

Multiple linear regression analysis showed that Pfirrmann grade was independently associated with FI in all three muscle groups. For each one-grade increase in Pfirrmann grade, SPL FI, SCap FI, and MF + SCer FI increased by an average of 6.480, 3.729, and 5.182 percentage points, respectively (SPL FI: *B* = 6.480, *P* < 0.001; SCap FI: *B* = 3.729, *P* < 0.001; MF + SCer FI: *B* = 5.182, *P* < 0.001; Table 6 and Figure 5). In addition, women had significantly higher SCap FI than men (*B* = 2.716, *P* = 0.041; Table 6), whereas the effect of sex on FI was not significant in the other muscle groups. Age was independently associated with MF + SCer FI (*B* = 0.218, *P* = 0.010; Table 6 and Figure 5). For CSA, sex showed the strongest association among the variables included in the model, with women showing significantly lower CSA than men. Because BMI was not included in the regression model, the CSA findings should be interpreted cautiously, as residual confounding related to body size cannot be excluded. After adjustment for sex and age, Pfirrmann grade was not significantly associated with CSA in any muscle group (SPL: *P* = 0.949; SCap: *P* = 0.151; MF + SCer: *P* = 0.925), whereas female sex was consistently associated with lower CSA (all *P* < 0.001; Table 6).

**Table 6:** Multiple linear regression analysis of paraspinal muscle parameters.

Dependent variable	Independent variable	*B*	Standardized *β*	95% CI	*P*
SPL FI	Sex, female vs. male	0.121	0.008	−1.925 to 2.166	0.907
SPL FI	Age	−0.054	−0.064	−0.173 to 0.066	0.377
SPL FI	Pfirrmann grade	6.480	0.768	5.244 to 7.716	<0.001
SPL CSA	Sex, female vs. male	−38.620	−0.354	−61.122 to −16.119	<0.001
SPL CSA	Age	−0.093	−0.014	−1.409 to 1.224	0.889
SPL CSA	Pfirrmann grade	0.439	0.007	−13.157 to 14.035	0.949
SCap FI	Sex, female vs. male	2.716	0.191	0.111 to 5.321	0.041
SCap FI	Age	0.081	0.096	−0.072 to 0.233	0.295
SCap FI	Pfirrmann grade	3.729	0.444	2.155 to 5.303	<0.001
SCap CSA	Sex, female vs. male	−48.684	−0.420	−70.966 to −26.403	<0.001
SCap CSA	Age	0.489	0.072	−0.815 to 1.793	0.458
SCap CSA	Pfirrmann grade	−9.819	−0.143	−23.283 to 3.644	0.151
MF + SCer FI	Sex, female vs. male	1.537	0.093	−1.263 to 4.337	0.278
MF + SCer FI	Age	0.218	0.222	0.054 to 0.382	0.010
MF + SCer FI	Pfirrmann grade	5.182	0.528	3.490 to 6.874	<0.001
MF + SCer CSA	Sex, female vs. male	−65.855	−0.534	−88.815 to −42.894	<0.001
MF + SCer CSA	Age	−0.387	−0.053	−1.731 to 0.956	0.568
MF + SCer CSA	Pfirrmann grade	−0.662	−0.009	−14.536 to 13.212	0.925

FI, fat infiltration; CSA, cross-sectional area; SPL, splenius capitis; SCap, semispinalis capitis; MF + SCer, multifidus plus semispinalis cervicis; CI, confidence interval. *B* represents the unstandardized regression coefficient. Sex, age, and Pfirrmann grade were included as covariates in each regression model. BMI was not included; therefore, CSA-related results should be interpreted with caution regarding potential body-size effects.

### 3.5 Differences in paraspinal muscle FI and CSA between PAC phenotypes

**Figure 6:**
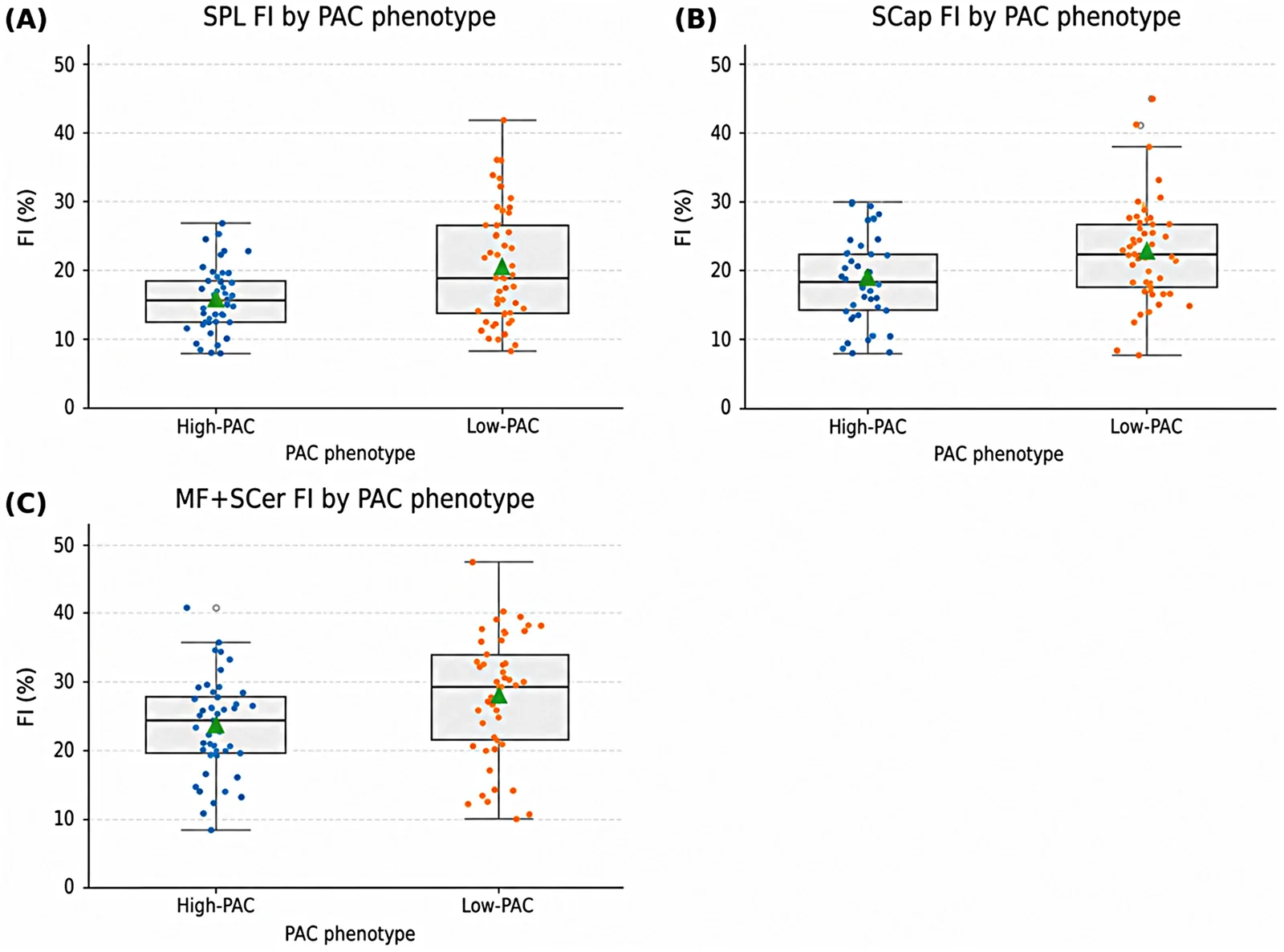
Comparison of paraspinal muscle FI between PAC phenotypes. **(A)** Comparison of SPL FI between PAC phenotypes (box plot with overlaid scatter points; the horizontal line within each box indicates the median, and the triangle indicates the mean). **(B)** Comparison of SCap FI between PAC phenotypes. **(C)** Comparison of MF + SCer FI between PAC phenotypes.

After stratification by PAC phenotype, FI in the SPL, SCap, and MF + SCer was significantly higher in the low-PAC/relatively stiff phenotype group than in the high-PAC/relatively mobile phenotype group. The differences were statistically significant for all three muscle groups (SPL FI: 20.512% ± 8.330% vs. 15.923% ± 4.664%, *P* = 0.001; Figure 6A; SCap FI: 22.824% ± 7.379% vs. 18.889% ± 6.310%, *P* = 0.007; Figure 6B; MF + SCer FI: 27.980% ± 8.919% vs. 23.638% ± 7.081%, *P* = 0.011; Figure 6C). After adjustment for age, sex, BMI, and Pfirrmann grade, the low-PAC/relatively stiff phenotype remained independently associated with higher FI in all three muscle groups (SPL FI: *B* = 2.553, 95% CI: 0.572 to 4.534, *P* = 0.012; SCap FI: *B* = 3.204, 95% CI: 0.687 to 5.721, *P* = 0.013; MF + SCer FI: *B* = 2.987, 95% CI: 0.238 to 5.736, *P* = 0.034). In contrast, CSA of the three muscle groups did not differ significantly between the two phenotype groups (*P* = 0.332, 0.273, and 0.358 for the SPL, SCap, and MF + SCer, respectively).

### 3.6 Stratified analysis of paraspinal muscle FI between PAC phenotypes according to Pfirrmann grade

To further reduce the influence of disc degeneration severity on the results, FI was compared between the high-PAC/relatively mobile group and the low-PAC/relatively stiff group within the same Pfirrmann grade. Among patients with Pfirrmann grade IV, in this exploratory stratified analysis, SPL FI was higher in the low-PAC/relatively stiff group than in the high-PAC/relatively mobile group (*n* = 16 vs. *n* = 11; 26.061% ± 5.345% vs. 19.285% ± 4.651%, *P* = 0.002; Table 7). Among patients with Pfirrmann grade III, SCap FI was also significantly higher in the low-PAC/relatively stiff group than in the high-PAC/relatively mobile group (*n* = 20 vs. *n* = 21; 23.702% ± 6.009% vs. 19.255% ± 6.434%, *P* = 0.028; Table 7). For MF + SCer, no significant between-group differences were observed within any Pfirrmann grade (all *P* > 0.05; Table 7).

**Table 7:** Comparison of FI between PAC phenotypes stratified by Pfirrmann grade.

Parameter	Pfirrmann grade	Low-PAC/Relatively Stiff Group, mean ± SD (n)	High-PAC/Relatively Mobile Group, mean ± SD (n)	*t*	*P*
SPL FI (%)	II	12.464 ± 3.152 (*n* = 8)	11.083 ± 2.596 (*n* = 12)	1.071	0.298
SPL FI (%)	III	15.878 ± 3.791 (*n* = 20)	16.778 ± 3.437 (*n* = 21)	−0.797	0.430
SPL FI (%)	IV	26.061 ± 5.345 (*n* = 16)	19.285 ± 4.651 (*n* = 11)	3.407	0.002*
SPL FI (%)	V	— (*n* = 5)	—(*n* = 1)	—	—
SCap FI (%)	II	14.961 ± 4.935 (*n* = 8)	13.788 ± 3.685 (*n* = 12)	0.610	0.550
SCap FI (%)	III	23.702 ± 6.009 (*n* = 20)	19.255 ± 6.434 (*n* = 21)	2.284	0.028*
SCap FI (%)	IV	24.108 ± 7.280 (*n* = 16)	22.746 ± 4.151 (*n* = 11)	0.559	0.581
SCap FI (%)	V	— (*n* = 5)	—(*n* = 1)	—	—
MF + SCer FI (%)	II	16.901 ± 5.948 (*n* = 8)	15.783 ± 4.645 (*n* = 12)	0.472	0.643
MF + SCer FI (%)	III	28.360 ± 7.657 (*n* = 20)	25.556 ± 6.120 (*n* = 21)	1.298	0.202
MF + SCer FI (%)	IV	30.969 ± 6.893 (*n* = 16)	27.555 ± 3.397 (*n* = 11)	1.515	0.142
MF + SCer FI (%)	V	— (*n* = 5)	—(*n* = 1)	—	—

The high-PAC/relatively mobile subgroup in Pfirrmann grade V included only one patient; therefore, between-group comparison was not performed.

FI, fat infiltration; PAC, posture-related angular change; SD, standard deviation; SPL, splenius capitis; SCap, semispinalis capitis; MF + SCer, multifidus plus semispinalis cervicis.

* Indicates *P* < 0.05.

## 4 Discussion

Conventional imaging assessment of cervical spondylosis mainly focuses on structural abnormalities of the intervertebral disc, bony elements, and neural compression. By contrast, imaging changes in the paraspinal muscles, which are active structures involved in segmental stability and postural control, are often less emphasized in clinical evaluation. Previous large-scale imaging studies have shown that C5–6 is one of the most commonly affected levels in cervical disc degeneration (1, 2). Therefore, the present study focused on single-level C5–6 disease and the corresponding paraspinal muscle changes. We found that FI in the SPL, SCap, and MF + SCer generally increased with higher Pfirrmann grades, whereas CSA did not differ significantly among grade groups. This pattern, characterized by increased FI without a parallel reduction in CSA, may suggest that changes in muscle composition occur earlier than, or independently of, changes in overall muscle size. It may also reflect partial replacement of atrophic muscle fibers by noncontractile fatty tissue. Local mechanical changes caused by disc degeneration, or reflex inhibition mediated by neural mechanisms, may reduce normal contractile stimulation of muscle fibers. At the same time, accumulation of fatty tissue within and between muscles may preserve the external muscle contour, making CSA less sensitive to early compositional change. Pinter et al. (16) reported that cervical paraspinal muscle fatty degeneration showed only minimal correlation with muscle CSA in patients undergoing anterior cervical discectomy and fusion, and recommended the use of validated fatty degeneration measures rather than simple area measurement in preoperative assessment. He et al. (17) found in a cohort of patients undergoing contiguous two-level cervical surgery that severe FI was associated with postoperative sagittal imbalance, particularly increased C2–7 sagittal vertical axis and abnormal segmental alignment, whereas vertebral body area-adjusted CSA was not significantly associated with these changes. Virk et al. (35) also demonstrated that cervical paraspinal muscle fat infiltration was related to cervical extension reserve, whereas muscle area alone was insufficient to explain this functional difference. These findings support the view that, in patients with single-level C5–6 cervical spondylosis, semi-quantitative FI measured on conventional MRI may be more sensitive than CSA for detecting changes in paraspinal muscle composition. However, because muscle strength, pain, neurological function, and patient-reported outcomes were not assessed, the clinical relevance of FI cannot be determined from the present data and should be evaluated in prospective studies incorporating clinical scales and functional testing (10, 13, 16, 17, 35–39).

After adjustment for sex and age, Pfirrmann grade remained independently associated with FI in all three muscle groups, whereas sex showed the strongest association with CSA among the variables included in the model. The regression results further support an association between the severity of disc degeneration and increased paraspinal muscle FI, independent of age and sex. However, because this was a cross-sectional study, the direction of causality cannot be determined. Several mechanisms may explain this association, including biomechanical compensation and local sterile inflammation. In the early stage of disc degeneration, segmental stability of the cervical spine may decline. To maintain local stability, the surrounding paraspinal muscles may remain under increased load or compensatory tension for a prolonged period. As compensatory capacity decreases, reduced dynamic contraction and relaxation may lead to disuse-related muscle fiber atrophy, followed by gradual fatty replacement. In addition, degenerated discs often show increased expression of inflammatory cytokines such as interleukin-1 and tumor necrosis factor-α (40). These inflammatory mediators may not only aggravate disc degeneration but also affect the surrounding paraspinal muscles by altering the local microenvironment. Activation of fibro-adipogenic progenitors and related signaling pathways has been implicated in muscle fat deposition and fibrosis (41–44). Taken together, disc degeneration, altered local mechanical loading, neuromuscular inhibition, and inflammatory microenvironmental changes may all contribute to increased paraspinal muscle FI. Nevertheless, inflammatory markers, electromyographic activity, and histological changes were not directly assessed in this study. Therefore, these mechanistic explanations remain speculative and should be verified in future prospective and mechanistic studies.

Further layer-specific analysis of the posterior cervical muscles showed that fatty change did not progress uniformly across different muscle layers. SPL FI showed a strong positive correlation with Pfirrmann grade, and significant differences were observed between most degeneration grades except between grades IV and V. This finding suggests that fatty change in the superficial splenius capitis may follow a relatively continuous stepwise pattern and may already be detectable during the transition from early to moderate degeneration. Therefore, SPL FI may be a sensitive cross-sectional imaging marker of muscle compositional changes related to local C5–6 degeneration. Its potential value for early identification and longitudinal monitoring requires confirmation in follow-up studies. In contrast, FI in the SCap and MF + SCer showed significant differences mainly in broader comparisons across degeneration grades, suggesting that different muscle layers do not respond to local degeneration in the same way. Total FI or a single deep-muscle measurement may therefore not fully represent the overall status of cervical paraspinal muscle degeneration. Regression analysis further showed that SPL FI had the strongest association with Pfirrmann grade in this study, suggesting that it may be particularly sensitive to local degenerative changes at C5–6. SCap FI was influenced by both sex and degeneration severity, indicating that sex-related differences should be considered when evaluating intermediate-layer muscles. MF + SCer FI was associated with both age and degeneration severity, suggesting that fatty change in the deep stabilizing muscles may reflect both local degeneration and age-related changes. Accordingly, imaging assessment of paraspinal muscles in single-level C5–6 cervical spondylosis should consider layer-specific FI changes across different muscle groups, rather than relying only on total FI or a single deep-muscle parameter to characterize the entire paraspinal muscle condition.

In addition to changes in muscle composition, this study also observed differences in the local C5–6 Cobb angle between supine MRI and standing lateral radiography in the same patient, reflecting posture-related angular change (18–23). Standing radiography more closely represents cervical alignment under weight-bearing conditions while maintaining a level gaze, whereas supine MRI is performed under non-weight-bearing conditions with support from the coil and headrest. Therefore, the angular difference between these two imaging positions may be influenced by loading status, body position, muscle control, and technical constraints of the imaging setup. Boudreau et al. showed that, in patients with cervical myelopathy, the mean Cobb angle measured on standing radiographs was significantly greater than that measured on supine MRI (11.6° vs. 9.2°), and 15.4% of patients who did not show lordosis on MRI showed lordosis on standing radiographs (18). van Santbrink et al. also reported that posture-related differences in cervical sagittal alignment may range from 1° to 16.6° (23). In addition, Zhou et al. reported correlations and reliability of cervical sagittal alignment parameters between plain radiographs and multipositional MRI images, while Paholpak et al. suggested that multipositional MRI could be used to evaluate angular parameters of the cervical spine. These findings indicate that posture-related or position-dependent angular changes may be reproducible to some extent and may have potential functional relevance (19, 20).

On this basis, the present study used an absolute PAC value of 5° as the prespecified classification threshold, where absolute PAC was defined as |standing radiographic Cobb angle − supine MRI Cobb angle|. Patients with |PAC| ≤ 5° were classified as having a low-PAC/relatively stiff phenotype, whereas those with |PAC| > 5° were classified as having a high-PAC/relatively mobile phenotype. This threshold was selected mainly on the basis of measurement error and clinical practicality. However, it remains an operational stratification criterion in this study and should not be regarded as a substitute for true segmental mobility assessed by dynamic flexion-extension radiographs (22, 29). Marques et al. (29) reported a standard error of 1.8° and a minimum detectable change of 5.0° for Cobb angle measurement. Sevin et al. (22) further found that lordosis measured on standing radiographs was, on average, 5.0° greater than that measured on supine MRI. Therefore, using 5° as the threshold may help reduce the influence of repeated measurement error on phenotype classification (22, 29). In the present study, FI in the SPL, SCap, and MF + SCer was significantly higher in the low-PAC/relatively stiff phenotype group than in the high-PAC/relatively mobile phenotype group. After stratification by Pfirrmann grade, between-group differences remained evident for SPL FI in grade IV and SCap FI in grade III.

These findings indicate a statistical association between higher FI and smaller PAC, suggesting that changes in paraspinal muscle composition may contribute to, or reflect, reduced angular adaptability of the affected segment across different positions and loading conditions. This relationship may be partly explained by several potential biomechanical mechanisms. First, the compensatory capacity of the superficial and intermediate muscle layers may decline. Anatomically and functionally, both the SCap and SPL contribute to cervical extension and postural maintenance. Increased FI in these muscles may reduce the amount of effective contractile tissue and impair active segmental adjustment across different postures. As FI increases, residual muscle fibers may become more prone to compensatory tension, while stability may depend increasingly on passive structures such as the facet joints and ligaments. Second, the fine stabilizing function of the deep muscle layer, including the MF and SCer, may be impaired. After fatty replacement occurs in these muscles, the central nervous system may maintain stability through co-contraction of larger peripheral muscles and greater reliance on passive bony and ligamentous structures. This reduction in active control may limit the range of angular adjustment during posture change. However, the present study cannot determine whether this represents a cause of muscle degeneration, a consequence of degeneration, or a parallel change accompanying disc degeneration. Finally, degeneration of the active muscular system and passive supporting structures may have additive effects. With increasing Pfirrmann grade, degenerative changes such as disc height loss and endplate sclerosis may further restrict segmental motion. Under these conditions, impaired paraspinal muscle function may further reduce postural adaptability of the segment, which may be reflected by decreased PAC and a low-PAC/relatively stiff phenotype (18–23, 35–39). The adjusted sensitivity analysis further suggested that the association between low PAC and higher FI was not fully explained by age, sex, BMI, or Pfirrmann grade, although the cross-sectional design still precludes causal inference.

Therefore, the relatively stiff phenotype defined in this study represents a composite imaging state that includes local structural degeneration, altered muscle composition, and reduced posture-related angular change. Previous studies have mostly discussed posture-related differences from the perspective of global C2–7 sagittal alignment. In contrast, the present study translated this common but often overlooked imaging phenomenon into an operational posture-related phenotype at the single C5–6 segment. PAC is essentially an operational measure based on the difference in local Cobb angle between different body positions and imaging modalities. It cannot replace dynamic flexion-extension radiography for assessing true segmental mobility. Its value lies in providing supplementary imaging information, under standardized measurement conditions, to describe angular differences of the affected segment between weight-bearing and non-weight-bearing states (18–23).

This study has several limitations. First, it was a single-center retrospective cross-sectional study. The patients were treated at a single institution, and their disease severity and treatment selection may differ from those of broader community-based populations. Therefore, selection bias may exist, the representativeness of the sample is limited, and causal relationships among disc degeneration, paraspinal muscle FI, and PAC cannot be determined. Second, the Pfirrmann grading system was originally developed for lumbar disc degeneration. Although it was applied in this study to describe C5–6 morphological degeneration with reference to previous cervical degeneration studies, there is still no universally accepted gold standard for grading cervical disc degeneration. In addition, the small number of patients with grade V degeneration may affect the stability of group comparisons and stratified analyses. Third, this study did not include a healthy control group and did not assess pain, neurological function, muscle strength, or cervical function using patient-reported questionnaires. Consequently, disease-related changes could not be fully distinguished from age-related changes, and the clinical relevance of FI and PAC in relation to symptoms and functional impairment could not be determined. Fourth, PAC was calculated from the difference in angular measurements obtained using two imaging modalities in different body positions. Although clinically practical, it cannot fully replace dynamic functional assessment. Fifth, this study used gray-level thresholding on conventional T2-weighted images for semi-quantitative FI assessment. This method allows relatively simple evaluation of muscle composition on routine clinical images, but it is not equivalent to quantitative fat fraction measurement obtained using Dixon or IDEAL-IQ sequences. Although reproducibility was improved by standardized PACS export settings, 8-bit grayscale images, scale calibration, a fixed threshold workflow, and reliability testing, threshold-based FI measurement may still be influenced by window settings and image export procedures. Therefore, these findings should be further validated in prospective cohorts using quantitative fat imaging.

## 5 Conclusion

In patients with single-level C5–6 cervical spondylosis, imaging changes in the paraspinal muscles were mainly characterized by increased FI, whereas CSA reduction was not evident. Compared with CSA, semi-quantitative FI measured on conventional MRI may be more sensitive for detecting changes in muscle composition, with SPL FI showing the strongest association with Pfirrmann grade. In addition, higher FI in the superficial and intermediate extensor muscles, particularly the SPL and SCap, was associated with the low-PAC/relatively stiff phenotype within comparable stages of disc degeneration. Combined assessment of PAC phenotype and paraspinal muscle FI may provide supplementary imaging information for describing angular differences of the affected segment between weight-bearing and non-weight-bearing conditions, and may support future imaging-based stratification and prospective validation studies.

## Data Availability

The raw data supporting the conclusions of this article will be made available by the authors, without undue reservation.
